# 4-Dimensional printing: exploring current and future capabilities in biomedical and healthcare systems—a Concise review

**DOI:** 10.3389/fbioe.2023.1251425

**Published:** 2023-08-22

**Authors:** Neha Agarwal, Vijendra Singh Solanki, Keshav Lalit Ameta, Virendra Kumar Yadav, Premlata Gupta, Shivraj Gangadhar Wanale, Ruchi Shrivastava, Anjali Soni, Dipak Kumar Sahoo, Ashish Patel

**Affiliations:** ^1^ Department of Chemistry, Navyug Kanya Mahavidyalaya, University of Lucknow, Lucknow, India; ^2^ Department of Chemistry, Institute of Science and Research (ISR), IPS Academy, Indore, India; ^3^ Centre for Applied Chemistry, School of Applied Material Sciences, Central University of Gujarat, Gujarat, India; ^4^ Department of Life Sciences, Hemchandracharya North Gujarat University, Patan, India; ^5^ School of Chemical Sciences, Swami Ramanand Teerth Marathwada University, Nanded, India; ^6^ Department of Chemistry, Medicaps University, Indore, India; ^7^ Department of Veterinary Clinical Sciences, College of Veterinary Medicine, Iowa State University, Ames, IA, United States

**Keywords:** 4-Dimensional Printing, sustainability, biomedical application, smart materials, pharmaceutical applications, healthcare, 4D bioprinting

## Abstract

4-Dimensional Printing (4DP) is the latest concept in the pharmacy and biomedical segment with enormous potential in dosage from personalization and medication designing, which adopts time as the fourth dimension, giving printed structures the flexibility to modify their morphology. It can be defined as the fabrication in morphology with the help of smart/intelligent materials like polymers that permit the final object to alter its properties, shape, or function in response to external stimuli such as heat, light, pH, and moisture. The applications of 4DP in biomedicines and healthcare are explored with a focus on tissue engineering, artificial organs, drug delivery, pharmaceutical and biomedical field, etc. In the medical treatments and pharmaceutical field 4DP is paving the way with unlimited potential applications; however, its mainstream use in healthcare and medical treatments is highly dependent on future developments and thorough research findings. Therefore, previous innovations with smart materials are likely to act as precursors of 4DP in many industries. This review highlights the most recent applications of 4DP technology and smart materials in biomedical and healthcare fields which can show a better perspective of 4DP applications in the future. However, in view of the existing limitations, major challenges of this technology must be addressed along with some suggestions for future research. We believe that the application of proper regulatory constraints with 4DP technology would pave the way for the next technological revolution in the biomedical and healthcare sectors.

## 1 Introduction

The term “4D Printing” (4DP) was coined firstly by Skylar Tibbits in 2014 (Tibbits, 2014) (Raviv D., 2014). Though this is just the inception to explore the potential of 3-Dimensional Printing (3DP) and 4DP in pharmaceuticals and healthcare, innovation is believed to be a non-stop process (Zema et al., 2017; Awad et al., 2018; Trenfield et al., 2018). Advancements are continuously being done in 3DP in overall potential including speed, accuracy, multiple materials, and fabrication accuracy. 3DP though has several applications in the biomedical field but also has some inherent weaknesses as it focuses on the manufacturing of static structures which are highly rigid in nature, retaining the shape of the originally printed structure and performing only a single function (Kuang et al., 2019; Wadher et al., 2021). Biomedical and Pharmaceutical applications require dynamic functions that cannot be fulfilled by 3DP technology. With the progression of biomedical nanotechnology (Gacem et al., 2022; Yadav et al., 2023b), researchers are now focusing on stimuli-responsive materials (smart polymers) which were beneficial in the development of drug delivery systems. These stimuli-responsive materials (smart materials) have the characteristic feature to respond to specific external stimuli when applied. These materials have the unique feature of showing pre-programmed responses and are therefore highly suitable for intelligent applications (Tan et al., 2019).

**FIGURE 1:**
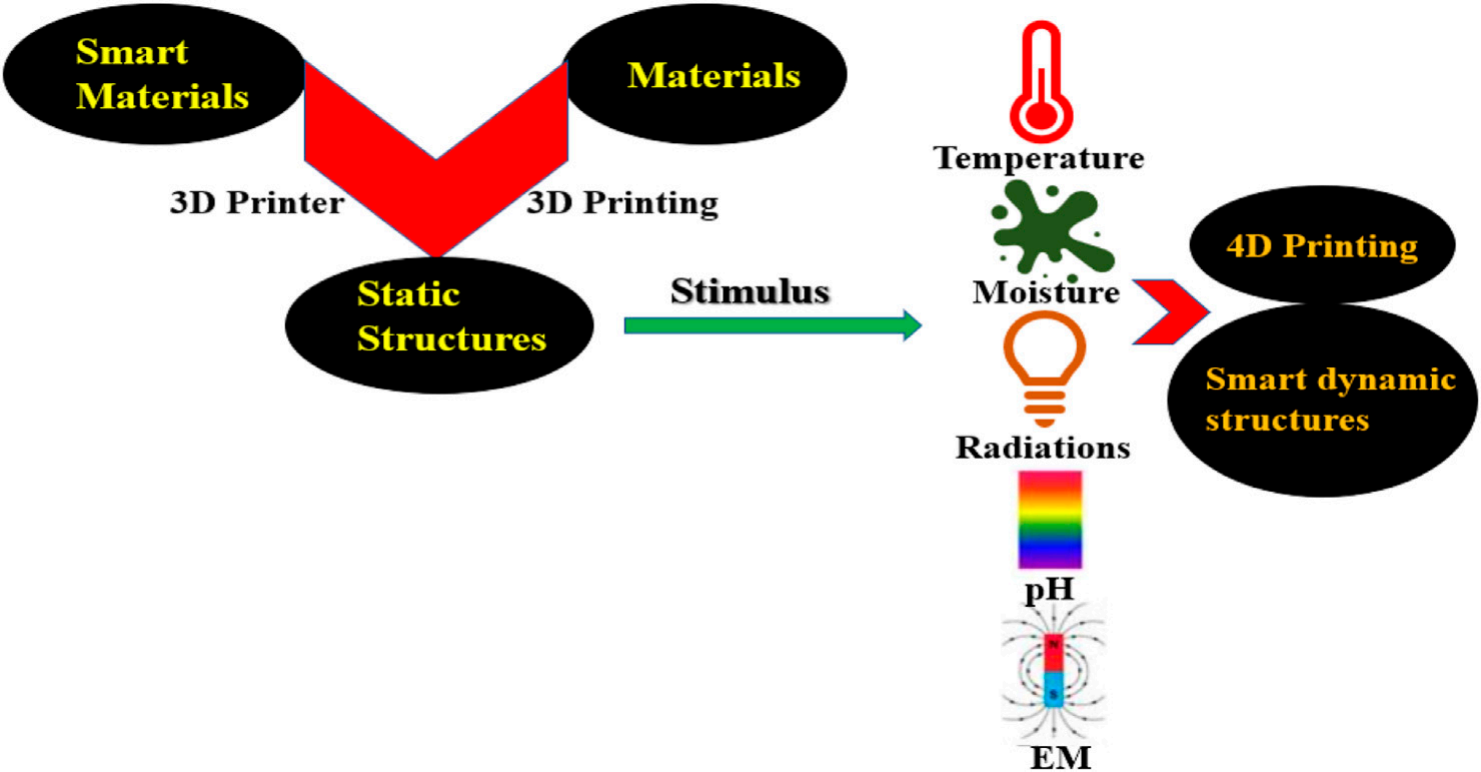
Conversion of 3D into 4D printing.

4DP is found to be the most remarkable transformation in the existing 3DP technique and is an exemplar of the additive manufacturing (AM) technique. 4DP is another smart technology to design flexible and dynamic structures and overcome the limitations of 3DP technology (Khoo et al., 2015). It is crucial to have information about 3DP, its history, and techniques of printing as it will help us to understand the latest concept of smart 4DP and its discovery in a better manner. Many simple and complex materials have been printed recently, by using Computer Aided Design (CAD) through 3DP technique under the overall control of a computer. 3DP has attained great interest among industries and academic institutions due to its high speed, great accuracy, and cheaper cost of production. 3DP is a multidisciplinary approach that collaborates various streams of sciences such as material, engineering science, mechanical & data processing, etc. 3DP is a unique technique by which complex structures can be designed which is not possible by conventional techniques (Melchels et al., 2012; Libonati et al., 2016; Choong et al., 2020; Nishiguchi et al., 2020). Successful discoveries have been done in the fields of remotely actuated robots (Maccurdy et al., 2016), bio-inspired designs (Wegst et al., 2015; Gu et al., 2017), micro-environments and tissues (Zhu et al., 2016) and drug delivery systems. 3DP has proven to be an intelligent and smart technology in the biomedical and other industrial sectors though 4DP is now in its infancy, but it has the super potential to transform the manufacturing industry in future. The basics of 4DP are depicted in Figure 1.

## 2 4D printing

A research group at MIT first conceptualized the idea of the 4DP of objects (Zhang Z. et al., 2019). The new era is getting more and more advanced due to progressive and continuous growth in different areas of science and technology. 4DP was found to be a dynamic shift in smart manufacturing techniques and is considered to be the most commendable transformation in the 3DP technique. Basically, 4DP can fabricate the 3DP materials by eternal stimuli. 4DP can create those products which can change their morphology in response to an external stimulus such as light, heat, temperature, pH, or water. The basic process involved in 3DP & 4DP techniques are very similar but the variation in 4D printed products is the use of smart materials for the design which is adaptable (Choi et al., 2015). This 4DP process demonstrates a quantum jump in AM smart techniques (André, 2017) which offers a medium to convert notions into reality with functionality that is driven by the performance. With the 4D technique, a large range of materials can be produced which are actively programmable and capable of self-transforming into another shape (Ahmed et al., 2021).

There are three salient features of 4DP that must be satisfied. First is the “selection of composite material” (or intelligent material) which should be responsive to external stimuli, when given. The second is the “external stimulus” which when added to the material makes the object animated. Last is the “time taken by the object to get stimulated” (Pei, 2014). Therefore, the 4DP method is an amazing addition of 3D printers, well-programmed design, and smart materials (Momeni et al., 2017; Shin et al., 2017; Wu et al., 2018). This technique is capable of reducing manufacturing labor costs to the minimum level. Massive products can be manufactured in 3D printers and only be used when exposed to the environment. The industry believes that surgical equipment, biomaterials, and nanotechnology are the most promising healthcare fields in which 4DP are most likely to be implemented (Hu et al., 2021). Latest advancements in the field of material science have produced many smart/intelligent polymeric materials for 4DP techniques that have the potential to self-assemble or deform themselves in response to an external stimulus such as UV light, temperature, magnetic and electric current, pH, etc. (Zhang et al., 2022).

There are many advantages of 4DP technology such as it is highly cost-efficient, time-efficient, less error-prone, highly productive, and sensitive (Tibbits, 2014). There is an increasing interest in studying 4DP technology in responsive structures such as printed actuators, soft robotics in medical devices (Bakarich et al., 2015), smart textiles, and aerospace (Fulcher et al., 2010). 4DP explores the latest branch from AM which explores the future possibilities for both 3DP and 4DP technologies.

## 3 Important factors required for 4D printing

**FIGURE 2:**
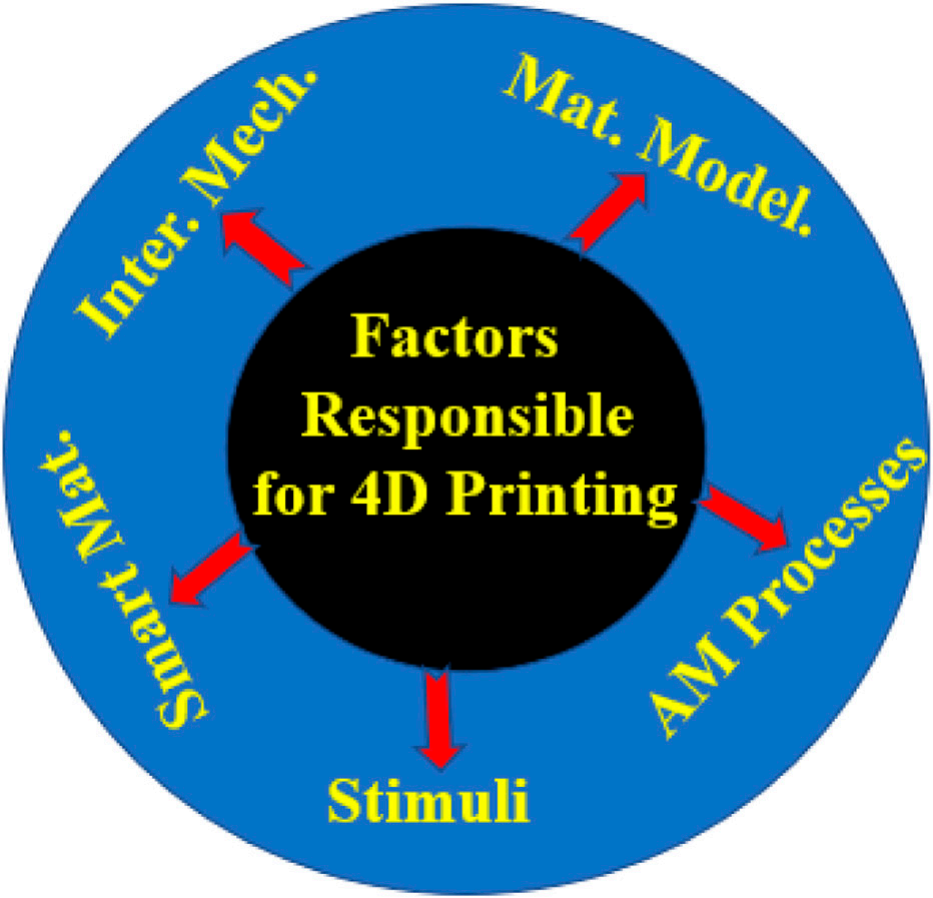
Factors responsible for 4DP.

4DP is highly dependent on five factors these are equipment/3D printers, stimulus-responsive material, stimuli, interaction mechanism, and the types of AM processes (Momeni et al., 2017; Zhang Z. et al., 2019). The AM process does not need any tool and it allows the production of printing material from the command received from the software. There are many types of AM processes such as Selective Laser Sintering (SLS), Selective Laser Melting (SLM), Stereolithography (SLA), Fused Deposition Modeling (FDM), Direct Ink Writing (DIW), Electron Beam Melting (EBM), etc. Most of the processes can print 4D material if, printing is in agreement with the printer. Another factor is the intelligent or smart stimuli-responsive materials used for advanced 4DP. The capability of the smart materials to self-transform themselves against external stimuli showed the strength of the 4DP technique. The next factor is the physical stimuli (temperature, light, moisture, magnetic energy (Yadav et al., 2020; 2023a), electric energy, Ultraviolet light (Modi et al., 2023b), etc., and chemical or biological) that are used in 4DP. The chemical stimuli cover the chemicals, pH level, etc. and the biological stimuli are the enzymes and glucose. Other factors are mathematical modeling and the mechanism of interaction. When an external stimulus is given to a programmable material it has the tendency to undergo the required transformation. It is important to plan the duration of mathematical modeling for which the stimulus will act on the programmable material. In Figure 2 all the factors are depicted which are responsible for 4DP.

## 4 Promising smart materials used for 4DP

**FIGURE 3:**
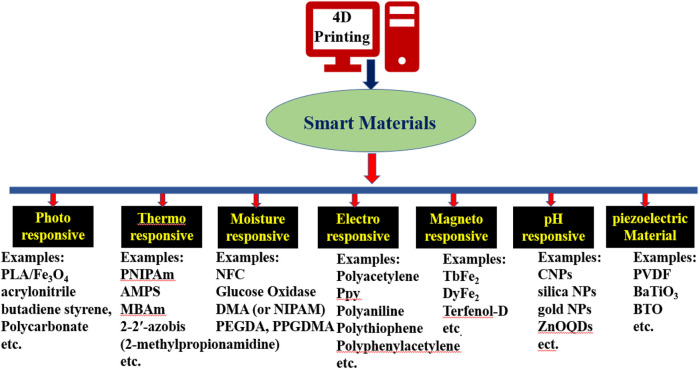
Stimuli-responsive smart materials used in 4D printing technology (Sun, 2015; Zhang F. et al., 2019; Farid et al., 2021; Chu et al., 2022). PLA, poly (lactic acid); PNIPAm or NIPAM: Poly (N-isopropylacrylamide); (AMPS), 2-Acrylamido-2-methylpropane sulfonic acid**;** MBAm, N,N′-Methylenebisacrylamide; NFC, nanofibrillated cellulose; DMA, Dimethylacetamide; PEGDA: poly (ethylene glycol) diacrylate; PPGDMA, poly (propylene dlycol) dimethacrylate;PPy, Polypyrrole; CNPs, Nanostructured carbon nanoparticles; ZNOQDs, surface-modified zinc oxide quantum dots; PVDF, piezoelectric polyvinylidene fluoride; BTO: Barium titanate.

Generally, the materials used for 4DP are called smart or intelligent materials because these are capable to change their properties with time (Leist and Zhou, 2016). Further intelligent materials are not defined universally and can sense the environmental stimulus and produce a useful response. This also includes conventional sensing materials such as piezoelectric materials which several researchers categorize as “intelligent” (Liu K. et al., 2021). Smart materials contribute an important and significant role in 4DP by acquiring, performing, and operating the given stimulus (Haleem et al., 2021). These sustainable materials respond to the given stimulus by performing the actuation, the shape-morphing, or functional modifications resulting in a complete change in the morphology (Invernizzi et al., 2018). Intelligent materials show unquestionable diverse features which can be exploited in products and structures such as responses to external stimuli like self-sensing, self-healing (automated recovery), self-actuating, self-diagnostic, and shape-changing (Liu et al., 2020). Therefore, smart or intelligent materials are those materials that provide a means of attaining an active and intelligent response in a product and have the potential to produce a myriad of increased functionalities. External stimuli are considered to be the biofuel of 4D printing technology (Pingale et al., 2023). A desirable response to external stimuli of the object is acquired by exploiting the physical properties of the printing materials. Therefore, the selection of smart material is highly dependent on the utility and employment of the object printed by 4DP (Ameta et al., 2022). Various smart materials and their applications have been discussed in Table 1 and Figure 3.

**TABLE 1:** Different variety of smart materials and important applications.

Smart materials	Description	Applications	References
Thermo-responsive smart materials	These involve the shape-memory effectwhich means the object regains its original shape after deformation and the shape-change effect. These materials are responsive to heat or temperature	Used widely in the medical field for the release of drugs and biomedical engineering	Sponchioni et al. (2019)
Photoresponsive smart materials	Light works as an indirect stimulus and when it is converted to heat it shows a response to light	Self-folding polymer sheets, self-assembled nanoparticles	Behl and Lendlein (2007), Habault et al. (2013), Liu et al. (2023b)
Moisture-responsive smart materials	These materials either release or absorb moisture with the change in relative humidity and cause deformation in the structure. Example-Hydrogels (it has high printability, and biocompatibility and are used as moisture-induced shape memory materials	Different varieties of hydrogels like natural and synthetic polymeric hydrogels and peptide hydrogels are applied in the 4DP	Osada and Matsuda (1995), Kopeček and Yang (2012), Shiblee et al. (2018), Erol et al. (2019)
Electro-responsive smart materials	Materials are responsive to electrical energy. Current is an indirect stimulus to show electro-responsive nature	For medical purposes, electro-simulative gels can be used, such as artificial muscle, sensors, actuators, and lenses, and as biomedical and soft materials	Ali et al. (2019)
Magneto-responsive materials	Materials responsive to magnetic energy are polymeric networks; functionalized chemically or physically with the magnetic nanoparticles including ferromagnetic and paramagnetic particles like iron, cobalt, nickel or their oxides. The first time the motion generation of austenite-martensite interfaces due to magnetic effect was reported where Fe (33.5)-Ni alloy was applied as an actuator and was justified by energy	Used in printed hydrogels micro-gripper. It also acts as a remote control for the magnetic material by applying a magnetic field	Ullakko (1996), Nichterwitz et al. (2021)
		Huge potential in material and polymer printing is also used to manipulate printed structures in a rapid manner	
pH-responsive materials	Due to the unique properties of these types of smart materials to work at different pHs of the organs in the body can be used as triggers in the drug delivery system	These types of smart materials have many biomedical applications in drug delivery, actuators and soft robots, valves, biocatalysts and stabilization of colloids	Jeong and Gutowska (2002), Soppimath et al. (2002), Schmaljohann (2006), Dai et al. (2008), Ali et al. (2019), Modi et al. (2023a)
Piezoelectric materials	These are other special properties of smart substances; such substances are sensitive to mechanical stress	Piezoelectric smart materials can be deformed under the influence of a mechanical force, therefore can also be utilized for the 4DP.	Nadgorny et al. (2016), Grinberg et al. (2019)

## 5 4DP applications

**FIGURE 4:**
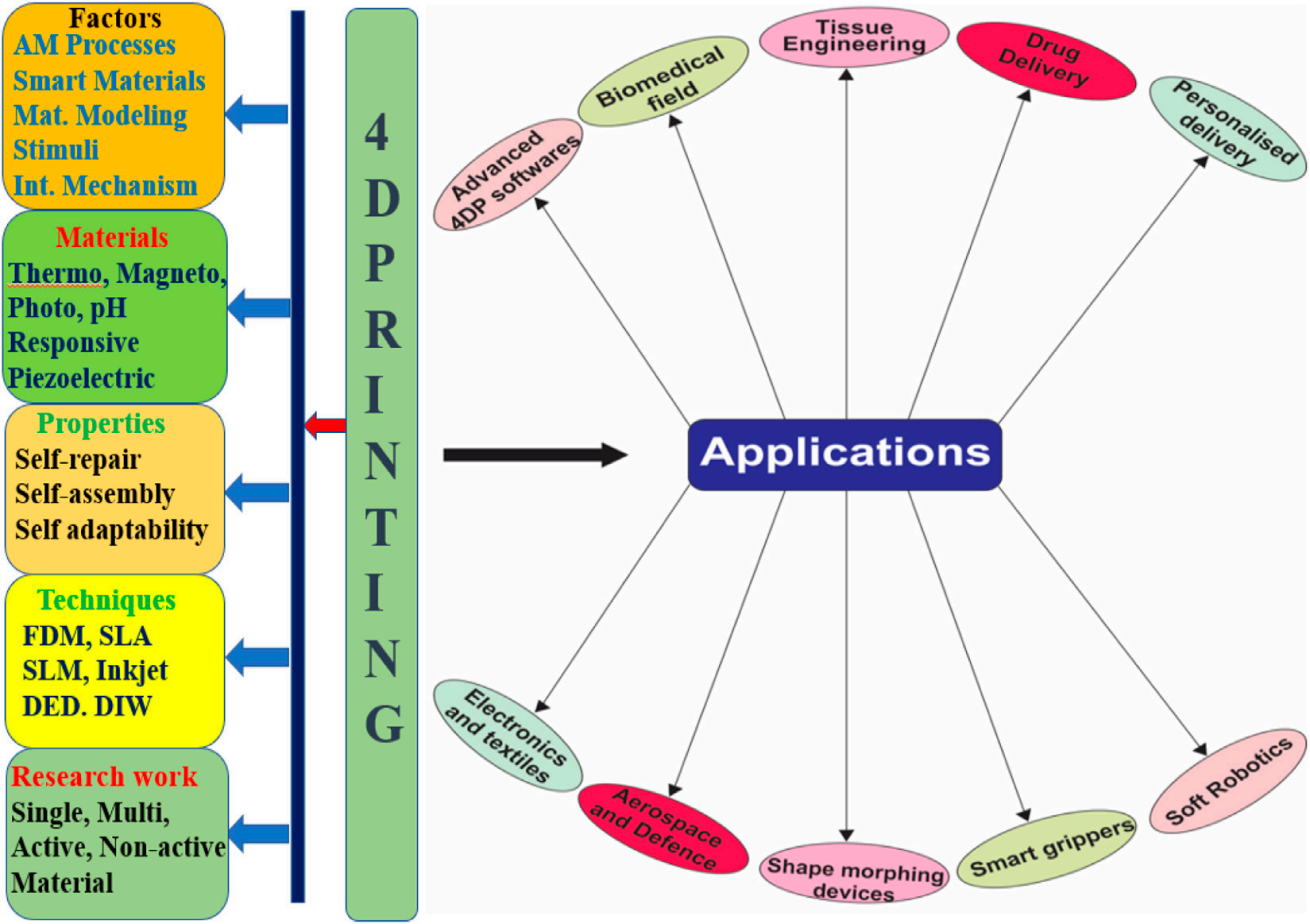
An overview of 4D printing technology and its applications. AM, Additive Manufacturing; FDM, Fused Deposition Modeling; SLA, stereolithography apparatus; SLM, Selective laser melting; DED, Direct energy deposition; DIW, Direct-Ink-Writing.

Many recent studies have explored a nascent field that integrates therapeutics with 3D and 4D printing. As a result, many formulation concepts and pharmaceutical devices have emerged that can be printed and possibly tailored to an individual. Traditional production is being replaced by rapidly expanding 4DP technology in a wide range of industries that includes medical engineering, healthcare (Yadav et al., 2022), automobile, electronics, textiles, aerospace engineering, defense (Yadav et al., 2023c), advanced 4DP software, etc. (Sahafnejad-Mohammadi et al., 2022). Different applications and a complete overview of 4DP are shown in Figure 4.

### 5.1 Opportunities in biomedical and healthcare

In medical devices and healthcare, a growing interest is seen in 4DP which explores a new arena of additive manufacturing research that has the potential to explore both 3DP and 4DP technologies. 4DP has a great advantage for diversified biomedical and healthcare applications, such as tissue engineering, bio-actuators, biosensors, and robotics (Liu B. et al., 2023). The medical and pharmaceutical sectors may be a potential field where 4DP objects can do miracles through biomedical applications of 4D printed objects dependent on chosen printing techniques, parameters, and the desired final usage (Choudhary et al., 2023a). Through a small incision, 4DP stents can be grafted into the body at the desired location, the shape can also be changed with the application of external stimuli and get useful results (Guo et al., 2023). 4D printing is a unique and developing smart technology that may add a novel approach to personalized medication designing and site-specific drug delivery concepts. This technique may take micro-robotics and bio-printing to a higher level and can surely make our healthcare and medical system better and smarter.

Biocompatibility is an important issue in the fabrication of Bio-Medical (BM) devices. Another issue of great importance is fabricating high-resolution structures that remain stable in their temporary as well as permanent spatial arrangements (Wang Q. et al., 2022). Due to the limitations in the physical properties, a single smart material can’t show a perfect structure but these materials are printed by smart 4DP techniques, resulting in their thermo-mechanical behaviors that can be created to promote controlled shape-memory behavior of the desired final printed structure (Ge et al., 2016). Autonomous shape memory and printability in response to an external stimulus are the most important requirements of 4DP materials (Shin et al., 2017). Kokkinis and his co-workers focused their work on 4DP which is magnetically triggered (Kokkinis et al., 2015).

It is said that the 4DP technique works on 3DP fabricating materials made from intelligent materials which are able to fold/unfold themselves. The use of these smart materials can be divided into two mechanisms, i.e., bio-adhesion and encapsulation based on drug delivery. There is a great global concern among scientists about novel drug delivery approaches and 4DP has the potential to offer the new concept of fabrication. In the search for real medical applications, still, high-quality research is needed to apply the technology to consumable medications (Choudhary et al., 2023b).

#### 5.1.1 Bioadhesion devices and drug delivery

An important application of 4D bio-printing is related to pharmaceuticals, bio-adhesion, and drug delivery systems where drugs or cells are encapsulated and then released under the influence of a particular stimulus (Trenfield et al., 2019). Efforts have been directed to develop responsive materials for being used in designing drug delivery systems for localized delivery of drugs and desired kinetics (Gazzaniga et al., 2023). The transdermal drug delivery system has greatly accelerated with microneedles that can penetrate transdermal delivery systems in a painless and convenient manner (Ma and Wu, 2017). A good example of painless drug administration through the skin was given by Liu et al., 2021. which has a hierarchical, limpet tooth-inspired architecture. Many stimuli-responsive systems can be used for targeted drug delivery and for achieving drug release, on demand. The surface properties of stimuli-responsive materials can also be modulated through intrinsic or extrinsic stimuli for enhancing penetration ability and improving cellular uptake (Li et al., 2019).

Bioadhesion devices are capable to initiate drug release by attaching themselves to the intestinal endothelium. According to a group of workers, this application develops a three-layered, mucoadhesive drug delivery system that is designed to work for sustained release formulation (Malachowski et al., 2014). Another bio-adhesive formulation using these materials is well-known multi-fingered thermo-responsive drug removal devices, also known as ‘theragrippers’ (Villar et al., 2011). Theragrippes are multilayered mini/microdevices with sharp microtips, which can latch onto the mucosal tissue in response to different kinds of *stimuli*. When exposed to temperatures above 32°C according to the predetermined design, it spontaneously grips onto the tissue as soon as introduced into the body at room temperature (Ghosh et al., 2020).

#### 5.1.2 Encapsulation devices

Encapsulation devices can be created for controlled drug delivery due to the unique feature of 4D printed materials to self-fold or unfold themselves. For example, scientists have printed a multisome when exposed to a certain pH; released its inner content and the signal was measured with fluorescence microscopy (Villar et al., 2011). A microrobot device has been created that consisted of a hydrogel bilayer which was fabricated by conventional lithography (Li et al., 2016). Pharmaceutical contents &l living cells can also be printed by 4DP as researchers have fabricated pancreatic beta-cell and fibroblasts onto structures (Azam et al., 2011).

#### 5.1.3 Biosensors, bioactuators and biorobotics

The 3D bioprinting (3DBP) technique is used to design biosensors (sensing devices in biological environments) previously. In recent years, implantable sensors have fetched revolutionary interest in healthcare that can provide continuous information for prolonged periods of time (Zhao et al., 2020). A transition from rigid and bulky devices to miniaturized flexible ones is seen with Hydrogels (as soft materials) to bridge the gap between soft biological systems and hard artificial machines (Ying and Liu, 2021).

A two-photon stereolithography approach has already been used to fabricate biosensors that are microcantilever-based, using polymers that are molecularly imprinted (Gomez et al., 2016) and the stereolithography technique has been used to make cantilevers that contain magnetic nonmaterial’s (Credi et al., 2016). These are useful to study the functions and nature of cells. 4DP bioactuators were investigated and fabricated by alginate hydrogel alginate hydrogels, which are able to withstand different temperatures (Bakarich et al., 2015). Another group printed a temporary shape airway stent which was converted back into a permanent shape by an increase in temperature (Zarek et al., 2017). 4DP has also got a great response in robotics and bioactuators which can be used for further advancement of smart technology. Various non-affordable materials can be fabricated by 4DP and used in the robotic industry. SM polyurethane micro-actuator with tunable properties was fabricated by a group of workers by 4DP (Hearon et al., 2015).

Self-healing pipes and hydrogels, and removing construction errors are some of the important applications of 4DP smart materials that will do wonders in the upcoming time. These 4DP materials are also used as sensors and printed artificial organs (Ahmed et al., 2021). Cells and tissues can be inserted into 4DP materials for providing mechanical forces for bioactuators/biorobots (Stanton et al., 2015). The biorobots can be used in drug delivery systems, difficult surgeries, and as therapeutic agents. In fact, certain difficulties and limitations in recent trials, such as gastric drug delivery systems, vascular stents, and muscle actuators have been worked out with 4DP (Yang et al., 2020). In orthopedics, 4DP can prove highly useable in the treatment of abnormalities due to their self-deformation.

#### 5.1.4 Medical applications

To accomplish biological applications; 3DBP is applied for the substance transfer method to fabricate various living materials. Fully customized, complex structures can be printed by transforming elaborated medical images like X-rays, computed tomography (CT) scans, and magnetic resonance imaging (MRI) scans into 3D Computer-aided design (3D CAD) models for the printing apparatus (Javaid and Haleem, 2019; Wang H. et al., 2022). Recently, bioprinting by 4DP is more adopted by surgeons, in organ transplantation; as a result, this field has developed tremendously (Lu et al., 2022). There are some limitations associated with current tissue engineering which automatically led to the progression of the latest techniques called 3DBP or 4DBP. Another highly approachable biomedical concept used a PCL-diacrylate-based polymer which can be applied to craniomaxillofacial bone defects (Arabiyat et al., 2021).

As compared to conventional tissue engineering; bioprinting has many benefits and most importantly high precision in cell deposing, and fabrication of tissues with high quantities & formed of large tissue-engineered objects. Basically, 4DBP includes a biomaterial and the maturation of the 3DP skeleton using the biomaterial (Kuang et al., 2019). Researchers have fabricated a polymeric grid-patterned grafted bone and glazed it with human nasal inferior turbinate tissue to facilitate graft degradation. Investigations demonstrated the improved osteoinductive and osteoconductive properties of the graft but the lesser mechanical strength of the synthetic graft was a challenge as compared to the natural bones. Therefore, more experiments and improvisation are required before a real-life application (Montgomery et al., 2017).

#### 5.1.5 Tissue engineering

3DP has fetched considerable attention in the biomedical field and tissue engineering (Kokkinis et al., 2015). Different kinds of biological structures (bone, liver tissue, blood vessels (Luan et al., 2022), heart issues, etc.) have been fabricated with 3DBP technologies (Yu et al., 2019). However, due to several limitations of 3DBP, the concept of 4DP has been applied in the field of tissue engineering. 3DP has been revolutionized by 4DP with shape and functional modification over time. The innovative 4DP technique has the potential to fabricate complex multilayer tissue constructs, thereby providing several advantages for tissue engineering and other applications (Miri et al., 2019). For tissue engineering applications a group of workers developed a new polycaprolactone (PCL) based formulation (Diedkova et al., 2023).

4DBP is required to attain such sprightly practice for designing critical and dynamic tissues, hierarchically (such as 4DBP, SM). Recent work presented the application of shape-memory scaffolds in the delivery of functional tissues that is minimally invasive (Gao et al., 2016). Another useful biomedical application of 4DBP in the rebirth of tissue and shipment of cells in a confined area in the body may be envisaged using the *in-situ* unfolding of 4DBP scaffolds. In the presence of biological moieties, the self-folding process can be done in response to external stimuli (Zhang and Lieber, 2016). To perform the delivery procedures in an accurate and wireless manner; biodegradable and bioelectronic devices can be integrated with scaffolds (Pati et al., 2015; Zhou et al., 2017). Similarly, cell traction forces have been utilized in another research work to make endothelialized tubes for 4D printed structures in a biomimetic way (Kuribayashi-Shigetomi et al., 2012). The potential benefits of 4DBP include high-resolution printing (different types of cells and excellent cell density tissues) and the capability to mass-produce tissue-engineering outputs (Chua and Yeong, 2015).

## 6 3D versus 4D: a comparative account

**FIGURE 5:**
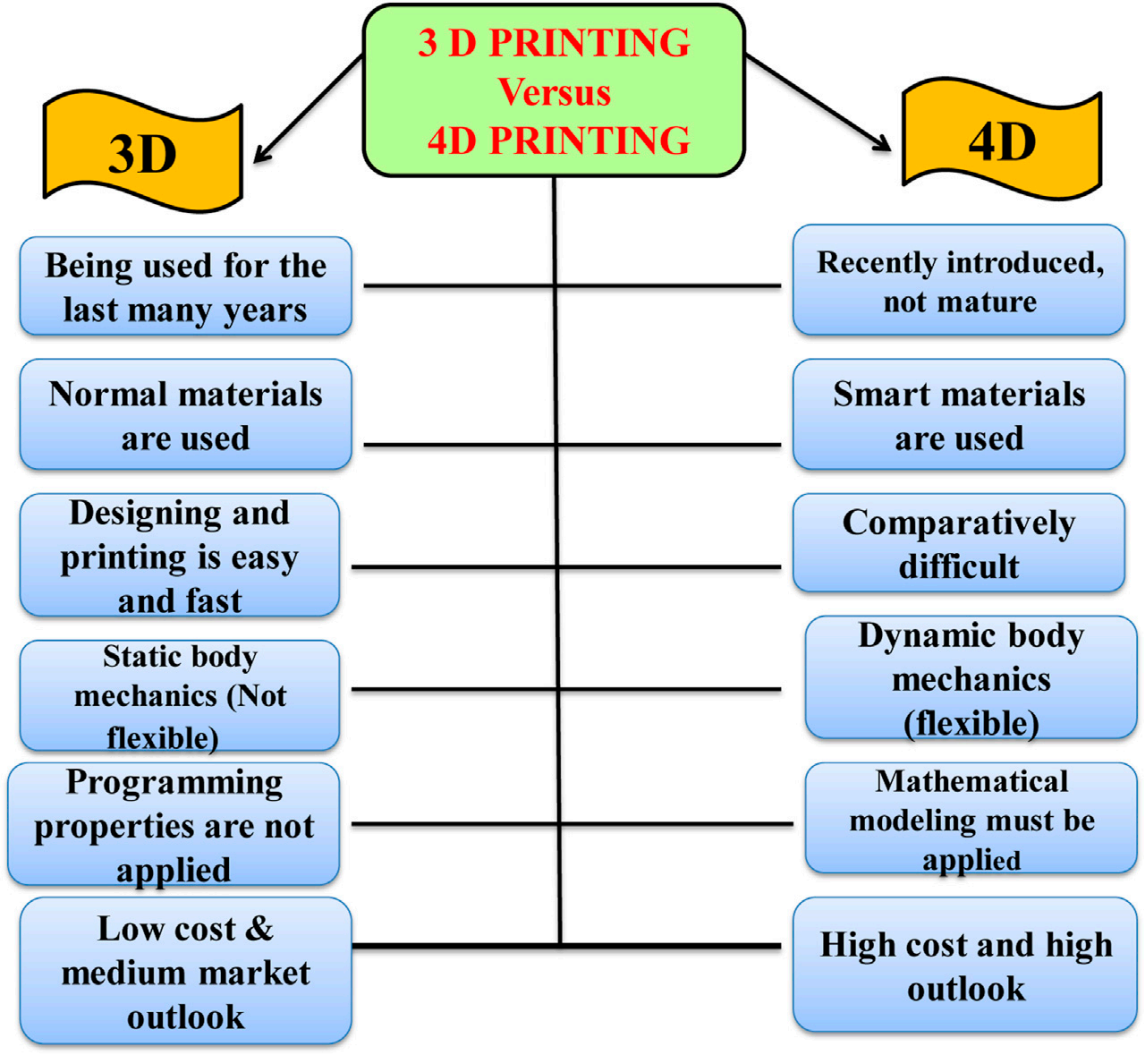
A comparative account of 3DP vs. 4DP.

The fundamental difference between 3DP and 4DP technologies occurs in the materials that are used to fabricate certain objects. Alternatively, 4DP has added mathematics, stimulus, and interaction parts using smart material, which is entirely different from 3DP. The requirements of dynamic structures and their relevant application such as soft grippers (Georgopoulou et al., 2021), self-assembled space antennae (Momeni et al., 2017), and self-healing polymers (Hager et al., 2010) couldn’t be met by the conventional 3DP technology which can only fabricate static structures from commercial filaments. 4DP can solve this bottleneck issue and offers advantages over 3DP in several aspects, which mainly depend on the fast growth of smart materials and multi-material structures (Jacobsen, 2016). Although 3DP and 4DP have similar advantages, 4DP outperforms 3DP in the dynamic status of produced items, hence, 4DP technology may be revolutionary next-generation of additive manufacturing in this regard (Mohanta, 2018). 4DP offers a change in the printed configuration in response to external stimulus over time, therefore, 4D printed structures should be fully preprogrammed using time as another dimension. Figure 5 summarizes a comparative account between 3DP and 4DP technologies.

## 7 Limitations of 4DP

Although, 3DBP has tremendous growth due to multiple applications and advantages (especially in medical and healthcare sector) but, it has certain limitations also. 3DPB considers only the original state of the printed structure and considers it to be lifeless (Gao et al., 2016). Still, 4DP is in the initial stage and require more research and development attention to bring the industrial and manufacturing revolution to the medical and pharmaceutical fields. Since 4DP technique is based on smart materials, hence, more production and physical & chemical properties should be studied (Khalid et al., 2022). The challenge and problems related to designing the organs and surgical instruments and their implantations and transformation in the body is really very difficult task. Current 3D printers can’t address fundamental 3DP issues such as simultaneous printing of polymers and metals, avoiding support structures, especially for inaccessible internal structures, high-cost printable materials, and slow print times. Therefore, in order to achieve the aims of 4DP, 3DP must be optimized on the basis of its use in 4DP. To address 3DP challenges, 5-axis 3D printing is currently of high interest (Zhang F. et al., 2019).

The lack of special software for 4DP is another significant limitation because most of the available software (such as design and slicing software) has been prepared for use in 3DP. To meet the requirements of 4DP, more powerful software has to be introduced. There are limitations in selecting smart materials that are used in the biological field like tissue engineering and tissue regeneration because of the limited number of intelligent materials that react to specific stimuli. Limited material availability, slow and inaccurate actuation, etc. are other challenges that need to be addressed. Though, 4DP technology has already proved its efficiency in multiple fields including medical engineering and with the development of inexpensive, high-accuracy printing devices, and most significantly, with the finding of novel smart bio-materials it is predicted to gain its top prospect soon.

## 8 4DP healthcare market

Technological advancements have brought revolutions in diagnostic and treatment methods. The growing need for advanced technology boosts investments in this field and is further propelling market growth. Consequently, the 4DP healthcare market is growing continuously and it is expected to reach USD 32.8 million in 2028 and CAGR will reach upto 26.7%. Different government and semi-government agencies are working and promoting this smart technology in various developed and developing countries. 4DP has wide applications in different sectors like automobiles, aviation, textiles (Patel et al., 2022), manufacturing industries, military operations, shoes, and fashion industries along with potential applications in healthcare and biomedical fields (Modi et al., 2022).

A driving factor for the use of 4DP in the healthcare field is the growing demand for organ transplants which requires functional implants in the area of tissue engineering and biomaterials. The enormous growth of 4DP is also due to its involvement in complicated cancer chemotherapies and surgical techniques due to the lesser use of surgical techniques (Feng et al., 2023). Also, targeted drug delivery systems use 4DP which could be utilized for delivering the mixture of pharmaceutical drugs in such a way that they act only on a targeted body site. Thus, the 4DP technology has paved the way for the medical field by making new inventions in drug delivery systems and improving the efficiency of medical treatments. It is rapidly replacing the conventional methods of manufacturing medical devices and offering greater functionality and advanced treatment (Melocchi et al., 2021).

## 9 Conclusion

4DP has grown tremendously since its inception and has proved its influence in different manufacturing & industrial sectors along with healthcare. Despite being a novel technology, 4DP has potential opportunities that are acknowledged by several experts in the field. 4DBP technology has gained lots of attention in biomedical and healthcare applications due to the development of stimulus-responsive biomaterials and tissue regeneration. Further advancement of 4DP will explore the tremendous applications in the biomedical field and would meet the upgraded medical requirements. The latest and highly effective methods of incorporating electronics into smart structures may result in the betterment of novel intelligent devices. Conclusively, 4DBP has an encouraging and hopeful future as a potential technology to copy traditional cellular structures. Therefore, it is expected that 4DP will certainly bring a bright future and revolution into different manufacturing and design industries and a paradigm shift to the plethora of undiscovered bigness that will make our future bright, smarter, and much more convenient. At the same time, more studies are required to resolve the existing challenges before 4DP serves as a powerful and a sustainable technique on a global level.
